# CO_2_ laser and/or fluoride enamel treatment against *in situ*/*ex vivo* erosive challenge

**DOI:** 10.1590/1678-775720150399

**Published:** 2016

**Authors:** Maísa Camillo JORDÃO, Gustavo Manzano FORTI, Ricardo Scarparo NAVARRO, Patrícia Moreira FREITAS, Heitor Marques HONÓRIO, Daniela RIOS

**Affiliations:** 1 - Universidade de São Paulo, Faculdade de Odontologia de Bauru, Departamento de Odontopediatria, Ortodontia e Saúde Coletiva, Bauru, SP, Brasil.; 2 - Universidade Camilo Castelo Branco, Departamento de Odontopediatria, São Paulo, SP, Brasil.; 3 - Universidade de São Paulo, Faculdade de Odontologia, Laboratório Especial de Laser em Odontologia, São Paulo, SP, Brasil.

**Keywords:** Dental enamel, Tooth erosion, Acidulated phosphate fluoride, Lasers

## Abstract

**Objective:**

This *in situ*/*ex vivo* study investigated the effect of CO_2_ laser irradiation and acidulated phosphate fluoride gel (APF) application, separately and in combination, on enamel resistance to erosion.

**Material and Methods:**

During 2 experimental 5-day crossover phases, 8 volunteers wore intraoral appliances containing bovine enamel blocks which were submitted to four groups: 1^st^ phase - control, untreated and CO_2_ laser irradiation, 2^nd^ phase - fluoride application and fluoride application before CO_2_ laser irradiation. Laser irradiation was performed at 10.6 µm wavelength, 5 µs pulse duration and 50 Hz frequency, with average power input and output of 2.3 W and 2.0 W, respectively (28.6 J/cm^2^). APF gel (1.23%F, pH 3.5) was applied on enamel surface with a microbrush and left on for 4 minutes. Then, the enamel blocks were fixed at the intraoral appliance level. The erosion was performed extraorally 4 times daily for 5 min in 150 mL of cola drink. Enamel loss was measured profilometrically after treatment and after the in situ phase. The data were tested using one-way Repeated Measures Anova and Tukey's test (p<0.05).

**Results:**

CO_2_ laser alone (2.00±0.39 µm) did not show any significantly preventive effect against enamel erosion when compared with the control group (2.41±1.20 µm). Fluoride treated enamel, associated (1.50±0.30 µm) or not (1.47±0.63 µm) with laser irradiation, significantly differed from the control.

**Conclusion:**

The APF application decreased enamel wear; however, CO_2_ laser irradiation did not enhance fluoride ability to reduce enamel wear.

## INTRODUCTION

Dental erosion is an increasing problem for the long-term health of the dentition^12^. Therefore, it is important to diagnose this condition as early as possible to initiate preventive measures^12^ ^,^ ^15^. However, it is difficult to control the different risk factors for the development of dental erosion, such as biological, behavioral and chemical factors^15^. Previous experiments showed several approaches for modifying the surface of the dental hard tissue so that erosive demineralization is reduced^10^ ^,^ ^25^. The use of fluoridated products shows only a slight preventive effect against erosion^10^ ^,^ ^13^ ^,^ ^14^. Therefore, other protective agents have been recently studied, namely those containing polyvalent metal ions, acid-protective layers application, products with added amino acids, peptides or proteins^3^ ^,^ ^13^ and laser irradiation.

CO_2_ laser, commercially available at the wavelength of 10.6 µm, produces radiation in the infrared regions that coincides closely with some of the apatite absorption bands, allowing dental surface changes^24^. The literature shows that these changes have promising results such as preventing dental caries^6^ ^,^ ^24^. Several studies have tested laser irradiation as an alternative approach for the prevention of dental erosion^11^ ^,^ ^19^ ^,^ ^22^ ^,^ ^23^ ^,^ ^26^. However, the results of the studies are controversial: some of them showed no effect^11^ ^,^ ^22^ ^,^ ^23^ ^,^ ^26^ while others pointed out good effects^5^ ^,^ ^17^ of CO_2_ laser irradiation in preventing erosion. In addition, CO_2_ laser irradiation enhances the efficacy of fluorides when considering dental caries^20^. Regarding erosion, there is no consensus about the synergistic effect between laser and fluoride^11^ ^,^ ^17^ ^,^ ^18^ ^,^ ^22^ ^,^ ^23^ ^,^ ^26^. The *in vitro* study, which found that laser alone was able to prevent enamel erosion and its protective effect was enhanced by fluoride application, used a commercially unavailable CO_2_ laser equipment ^17^. Thus, we need to clarify whether is possible to use the CO_2_ laser to prevent erosion on equipment available to the clinical dental practitioner.

A different protocol that better simulates the presence of acquired pellicle and the action of saliva^28^, which is in the *in situ* ones, could have a different impact on the effect of laser in dental erosion. Especially because the homeostatic oral mechanism against erosion will be partially present, diminishing the erosive challenge.

Considering these aspects, this study was designed to evaluate the effect of CO_2_ laser-irradiation associated or not with acidulated phosphate fluoride gel (APF) application on enamel resistance to erosive challenge.

## MATERIAL AND METHODS

### Ethical aspects

Eight healthy adult volunteers (6 female, 2 male, aged 19–23 years) who fulfilled the inclusion criteria (physiological salivary flow rates- stimulated: 7.53 mL/min, pH 7.1; unstimulated: 1.92 mL/min, pH 6.9; good oral health: no frank cavities or significant gingivitis/periodontitis) without violating the exclusion criteria (systemic illness, pregnancy or breastfeeding, use of fixed or removable orthodontic appliances, hyposalivation) were enrolled following CONSORT guidelines. The study conformed to the Declaration of Helsinki and was performed to the guidelines of good clinical practice. Ethical approval for the study involving human subjects was granted by the local Ethics Committee (Process 008-2009). The study was planned as a prospective, single center, double blind, and two-cell study with an overall experimental period of 2x 5 days (washout period of 7 days). The volunteers were the experimental units and the study variable consisted of a three-level treatment (CO_2_ laser irradiation, fluoride application and fluoride application before CO_2_ laser). Participants received written instructions and were extensively trained for all procedures. Informed consent was obtained from all volunteers before the study.

### Sample preparation

**Figure 1:**
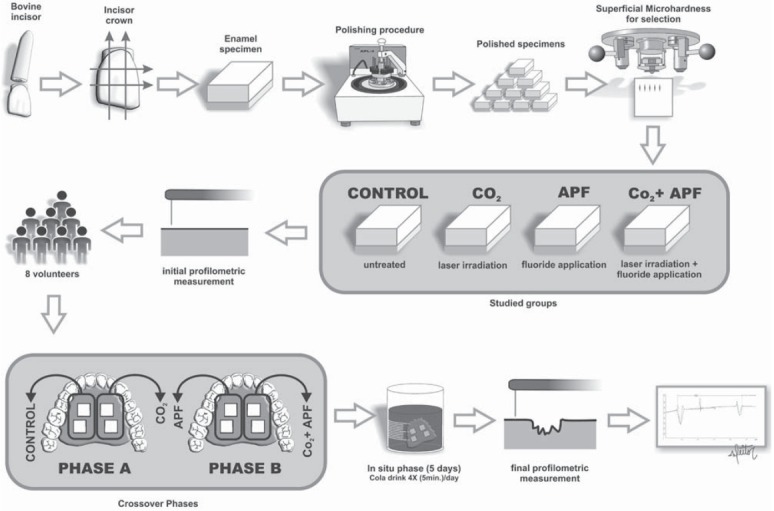
Flowchart of the experimental design

Bovine enamel blocks (4x4x3 mm) were prepared from extracted bovine incisors, which had been previously stored in 2% formaldehyde solution (pH 7.0) for 30 days at room temperature. One block was cut from each crown using a cutting machine (Isomet Low Speed Saw, Buehler Ltd., Lake Bluff, Illinois, USA) and two diamond disks (Extec Corp., Enfield, CT, USA), which were separated by a 4 mm thickness spacer. The enamel surface was ground flat with water-cooled silicon carbide discs (320, 600 and 1200 grades of Al_2_O_3_ papers; Buehler Ltd., Lake Bluff, Illinois, USA) and polished with felt paper wet by diamond spray (1 µm; Buehler Ltd., Lake Bluff, Illinois, USA). This procedure resulted in a removal of about 200 µm depth of enamel, which was controlled by a micrometer. The surface hardness was determined by performing five indentations (Knoop diamond, 25 g, 5 s, HMV-2000; Shimadzu Corporation, Tokyo, Honshu, Japan) for selection purpose. Sixty four enamel blocks with hardness ranging from 320 to 364 KHN (335.8±3.6) were numbered in an ascending order and randomly distributed into 8 volunteers (n=8) and 4 groups (with 2 blocks *per* group). The enamel blocks were sterilized by exposure to ethylene oxide gas before the treatment. The groups were tested during two phases: 1^st^ phase - (C) control, untreated blocks and (L) CO_2_ blocks, treated with laser irradiation; 2^nd^ phase - (F) fluoride blocks, treated with acidulated phosphate fluoride gel (APF, 1.23% fluoride) application and (F+L) fluoride and CO_2_ blocks, treated with APF application before CO_2_ laser irradiation. The experimental design is presented in Figure 1.

Before the experiment, two layers of nail varnish were applied to two thirds of the surface of each sample to maintain reference surfaces to determine lesion depth after the experiment. Each 4 samples (2 *per* group) were fixed with wax into the recesses of the individual acrylic palatal appliances. The position of each group in the appliance was randomly determined (Excel, Microsoft, Redmond, Washington, USA).

### Fluoride and CO2 laser treatment

Laser irradiation was performed with a commercially available CO_2_ laser (UM-L30, Union Medical Engineering Co., Seoul, Korea) at 10.6 µm wavelength, 5 µs pulse duration, 50 Hz frequency and spatial mode TEM00. One trained dentist irradiated the exposed surface of the samples (free from nail varnish) for 15 to 20 s by moving the laser probe tip continuously with a special device to standardize the distance of 10 mm from the sample surface (Beam diameter: 0.3 mm; Spot size: 7x10^-4^ cm^2^). Samples were irradiated with average power input and output of 2.3 W and 2.0 W, respectively (Energy/pulse of 1 J and Energy density of 0.14 J/cm^2^). For fluoride treatment, the acidulated phosphate fluoride gel (pH 3.5, Flugel, DFL, Rio de Janeiro, RJ, Brazil) was applied on each enamel surface with microbrush and left on for 4 minutes. After the time elapsed, the excess gel was removed with cotton swabs. In group F+L, the laser irradiation was performed immediately after the excess gel was removed with cotton swabs.

Two days after treatment (blocks were maintained in 100% humidity), enamel blocks were subjected to the *in situ* phase. This period was necessary for profilometric measurement after treatment and for the inclusion of the blocks in the palatal appliance.

### *In situ/ex vivo* experiment

Ten days before and throughout the entire experiment, the volunteers brushed their teeth with fluoridated toothpaste (Colgate Tripla Ação, Colgate-Palmolive, São Bernardo do Campo, São Paulo, Brazil). The volunteers wore the appliance for 12 h before the formation of a salivary pellicle. After the 12 h lead-in period, the erosive challenge was performed four times daily *ex vivo* (morning, midday, afternoon and evening) by immersing the appliance in a cup containing 150 mL freshly opened bottles of Coca-Cola (pH 2.6, 0.32 ppm F, Coca-Cola Company, Marília, São Paulo, Brazil) at room temperature for 5 minutes, with at least 3 h apart. Immediately after erosion, the appliances were reinserted into the mouth. The appliances were worn day and night, except during meals and oral hygiene procedures (4 times daily, 1 h each) when they were wrapped in wet gauze and stored in a closed box. The participants were advised not to eat or drink wearing the appliances.

### Profilometric analysis

Enamel loss was quantitatively determined using a contact profilometry (Hommel Tester T1000, VS, Schwenningen, Baden-Württemberg, Germany) after fluoride application and/or CO_2_ laser irradiation (first measurement), and after the *in situ* phase (final measurement). For profilometric measurement, the nail varnish was carefully removed by mechanical displacement movement with a spatula on the enamel border. Then the diamond stylus moved from the reference to the exposed area (length of profile: 1.5 mm). Five profile measurements were performed in the center of each sample and averaged. The reproducibility of the first and final measurements (tenfold tracing of one sample) was ±0.05 and ±0.1 μm respectively. The repeat analysis of one trace showed a standard deviation of ±0.02 μm.

After the first profilometric measurement, the reference area of the specimens was again covered with nail varnish. To assure that the nail varnish was placed over the original reference area, the position of the nail varnish was marked by carving with a scalpel blade at the borders of the sample.

### Statistical analysis

The assumptions of equality of variances and normal distribution of errors were checked for all the variables tested using the Bartlett and Kolmogorov–Smirnov tests (SigmaPlot for Windows version 11.0, Erkrath Germany, North Rhine-Westphalia, Germany), respectively. The assumptions were satisfied and one-way Repeated Measures Analysis of Variance was applied, followed by Tukey’s test (SigmaPlot for Windows version 11.0, Erkrath Germany, North Rhine-Westphalia, Germany). The significance level was set at 5%.

## RESULTS

Table 1 shows enamel loss [mean±standard deviation (µm)] after treatment, after the *in situ*/*ex vivo* phase and the difference between them. All treatments induced minimal enamel loss and undesired effects as craters or crack were not visible on specimens after laser irradiation. Data revealed that for cumulative enamel loss (treatment+*in situ/ex vivo* phase) and also for the amount of it caused by the erosive challenge alone (the difference between after treatment and the *in situ* phase), the acidulated phosphate fluoride gel individually (F) or associated to laser irradiation (F+L) significantly differed from the control (C). Laser irradiated group showed similar enamel loss when compared with control and with all experimental groups (F and F+L).

**Table 1:** Enamel loss (µm) in the different groups after treatment with fluoride (APF) and/or carbon dioxide (CO2) laser, after erosive challenge *in situ*/*ex vivo* and the difference between after treatment and erosive challenge

Loss	Control (C)	Laser (L)	Fluoride (F)	Fluoride+Laser (F+L)
Pretreatment	0^a^	0.19±0.13^b^	0.11±0.05^b^	0.12±0.06^b^
*In situ*	2.41±1.20^a^	2.00±0.39^a,c^	1.47±0.63^b,c^	1.50±0.30^b,c^
Difference	2.41±1.20^a^	1.77±0.43^a,c^	1.34±0.62^b,c^	1.38±0.31^b,c^

Different letters mean statistical differences between columns (Repeated Measures Analysis of Variance /Tukey’s test, p<0.05)

## DISCUSSION

This study evaluated the effect of CO_2_ laser therapy with or without acidulated phosphate fluoride gel application as preventive measures against erosion. Since erosion is influenced by biological factors, such as saliva and acquired pellicle^9^, this study was conducted *in situ*/*ex vivo*, to get the most reliable results.

Commercially available CO_2_ lasers are based on the wavelength of 10.6 µm and can be adapted to operate at the other wavelengths^8^. The absorption coefficient for dental enamel at wavelength of 9.6 µm is ten times higher than for the wavelength of 10.6 µm^7^, which would result in an effective energy deposition in the outer enamel layer, with minor risk of pulp damage. On the other hand, the 10.6 µm wavelength CO_2_ laser presents an enamel absorption depth 11 µm higher^7^, resulting in a thicker layer of enamel modification, whose characteristics will be presented later as theories for the ability of laser irradiation to decrease enamel solubility. This thicker layer of modified enamel is required for the long term prevention of dental erosion. Though the emission at 10.6 µm was used in this study, the irradiation was performed with short pulse to minimize the cumulative energy deposition, which determines the rise in pulp temperature. The laser parameters vary among studies and there is no consensus about the best one, thus a parameter similar to previous erosion *in vitro* study^26^ was used. The choice for the fluoride application before laser irradiation is also in accordance with another study that showed better anti-erosive effect with Nd:YAG laser^19^. Alterations on enamel surface due to the treatment were considered, since the nail varnish applied to reference area, for lesion depth determination, was established before laser or fluoride treatment, and enamel loss was evaluated. The studied treatments induced probably none enamel loss (<0.2 µm), because the dissolution of the enamel due to erosive attack leads to surface roughening of about 0.4 µm, therefore enamel alterations below 0.4 µm are generally not considered as enamel loss^1^. The treated enamel alteration did not interfere with the present results, since the cumulative enamel loss after treatment, additionally to the *in situ* phase, showed similar differences among the groups when compared with enamel loss after the erosive challenge alone (the difference between after treatment and the *in situ* phase).

This study showed that CO_2_ laser, which provides a very efficient interaction with dental tissues and is expected to present preventive effect against erosion, did not diminish enamel loss *in situ*. Wiegand, et al.^26^ (2010) using similar parameters and Steiner-Oliveira, et al.^23^ (2010) also failed to show protective effect of CO_2_ laser irradiation against erosion *in vitro*. Three theories have been proposed for the ability of laser irradiation to decrease enamel solubility against dental caries and they are all related to the increase of the tissue’s temperature. The physical seal achieved by melting the surface thought partial fusion and recrystallization of enamel prisms is considered one of the theories^8^. However, previous study indicates that the fusion of enamel does not inhibit permeability^2^. The second theory is related to crystallographic changes, resulting in the formation of pyrophosphate and carbonate loss, which might decrease the solubility of hydroxyapatite^8^. In cases of erosive challenge when the pH of the erosive acid is below approximately 3.9, the solution will be undersaturated regarding the enamel mineral. In other words, the erosive solution will most probably dissolve the enamel regardless of its fluoride, phosphate or calcium concentration^12^. A third theory is the decomposition of the organic matrix, since the matrix decomposition products (globular precipitates) can seal the enamel pores, blocking the entrance of acid ions and forming mineral reservoir^8^. Probably, in this study, these globular precipitates were not able to inhibit the contact between erosive acid and enamel. The hypothesis to explain such difference in the laser effect between dental caries and erosion might be related to the fact that the acid challenges involved in the erosive process are more aggressive than those in the carious process, leading to fast enamel dissolution.

The APF application group promoted enamel resistance against enamel erosion, which is in accordance with the literature^10^ ^,^ ^19^ ^,^ ^23^. It is well established that the application of fluoride results in the formation of a calcium fluoride (CaF_2_)-layer, especially when the product has a low pH and a high fluoride concentration^3^ ^,^ ^14^ ^,^ ^21^. This layer is assumed to behave as a physical barrier hampering the contact between the acid and the underlying enamel or to act as a mineral reservoir, which is attacked by the erosive challenge.

Contrary to this study, Ramalho, et al.^17^ (2013) found that laser alone showed significant erosion prevention (52%) and the percentage of reduction compared with the control group was higher (73%) when the CO_2_ laser was associated with fluoride. However, the frequency of erosive challenge was twice lower. On the other hand, other studies showed similar results to this study^11^ ^,^ ^22^. Mechanisms of combined effect of fluoride and laser remained unclear. It is suggested that laser plus fluoride enhance calcium fluoride like (CaF_2_)-layer formation^29^ and other suggests that laser transforms hydroxyapatite into fluorapatite in the presence of fluoride^16^. Some authors also defend the hypothesis that the association changes the enamel’s permeability, and the laser can change the organic matrix present in the enamel and promote a blockage of inter- and intra-prismatic spaces with compromised diffusion of ions^4^ ^,^ ^27^. In this study, although the preventive effect of the association between fluoride and laser was observed compared with the control group, no synergistic effect could be found. The hypothesis for this result is that only APF application protected enamel against erosion by the formation of a calcium fluoride layer and CO_2_ laser irradiation did not enhance or influence its efficacy. In addition, when considering dental erosion, the formation of fluorapatite has minor preventive effect since the erosive acid readily dissolves fluorapatite^12^. Similar results were observed *in vitro* ^11^ ^,^ ^18^ ^,^ ^22^ ^,^ ^23^. However, the comparison among studies cannot be appropriately done since different laser equipment/parameters and also different experimental erosion models were used.

## CONCLUSION

In conclusion, within the limits of this *in situ*/*ex vivo* study, the results suggest that the APF application or its association to laser irradiation decreased enamel loss. CO_2_ laser irradiation did not prevent enamel erosion compared with the control group; however, it showed similar enamel loss when compared with APF and its association to laser. Considering the cost-benefit and the availability of treatment for patients at risk of enamel erosion, fluoride is still the most feasible preventive measure.
